# Use of a gene expression signature to identify trimetazidine for repurposing to treat bipolar depression

**DOI:** 10.1111/bdi.13319

**Published:** 2023-03-23

**Authors:** Chiara C. Bortolasci, Srisaiyini Kidnapillai, Briana Spolding, Trang T. T. Truong, Timothy Connor, Courtney Swinton, Bruna Panizzutti, Zoe S. J. Liu, Andrew Sanigorski, Olivia M. Dean, Tamsyn Crowley, Mark Richardson, Kiymet Bozaoglu, Katerina Vlahos, Stephanie Cowdery, Brad Watmuff, Stephan F. Steyn, De Wet Wolmarans, Barend J. Engelbrecht, Christina Perry, Katherine Drummond, Terence Pang, Stéphane Jamain, Laura Gray, Sean L. McGee, Brian H. Harvey, Jee Hyun Kim, Marion Leboyer, Michael Berk, Ken Walder

**Affiliations:** ^1^ IMPACT The Institute for Mental and Physical Health and Clinical Translation, School of Medicine, Deakin University Geelong Australia; ^2^ The Florey Institute of Neuroscience and Mental Health Parkville Australia; ^3^ Bioinformatics Core Research Facility (BCRF) Deakin University Geelong Australia; ^4^ Murdoch Children's Research Institute Parkville Victoria Australia; ^5^ Department of Paediatrics University of Melbourne Parkville Victoria Australia; ^6^ Centre of Excellence for Pharmaceutical Sciences, Faculty of Health Sciences North‐West University Potchefstroom South Africa; ^7^ Univ Paris Est Créteil, INSERM, IMRB, Translational Neuropsychiatry, AP‐HP, DMU IMPACT, FHU ADAPT Fondation FondaMental Créteil France; ^8^ SAMRC Unit on Risk and Resilience in Mental Disorders, Department of Psychiatry and Mental Health and Neuroscience Institute University of Cape Town Cape Town South Africa; ^9^ Orygen, The National Centre of Excellence in Youth Mental Health Parkville Australia

**Keywords:** bipolar depression, drug repurposing, gene expression signature, trimetazidine

## Abstract

**Objectives:**

The aim of this study was to repurpose a drug for the treatment of bipolar depression.

**Methods:**

A gene expression signature representing the overall transcriptomic effects of a cocktail of drugs widely prescribed to treat bipolar disorder was generated using human neuronal‐like (NT2‐N) cells. A compound library of 960 approved, off‐patent drugs were then screened to identify those drugs that affect transcription most similar to the effects of the bipolar depression drug cocktail. For mechanistic studies, peripheral blood mononuclear cells were obtained from a healthy subject and reprogrammed into induced pluripotent stem cells, which were then differentiated into co‐cultured neurons and astrocytes. Efficacy studies were conducted in two animal models of depressive‐like behaviours (Flinders Sensitive Line rats and social isolation with chronic restraint stress rats).

**Results:**

The screen identified trimetazidine as a potential drug for repurposing. Trimetazidine alters metabolic processes to increase ATP production, which is thought to be deficient in bipolar depression. We showed that trimetazidine increased mitochondrial respiration in cultured human neuronal‐like cells. Transcriptomic analysis in induced pluripotent stem cell‐derived neuron/astrocyte co‐cultures suggested additional mechanisms of action via the focal adhesion and MAPK signalling pathways. In two different rodent models of depressive‐like behaviours, trimetazidine exhibited antidepressant‐like activity with reduced anhedonia and reduced immobility in the forced swim test.

**Conclusion:**

Collectively our data support the repurposing of trimetazidine for the treatment of bipolar depression.

## 1 INTRODUCTION

Many medications for BD have substantial tolerability burdens, and do not adequately alleviate symptoms for many people with BD, particularly depression^1^ that leaves individuals severely incapacitated. Undesirable side effects also lead to high levels of treatment non‐adherence. The first line treatment for BD is a mood stabiliser such as lithium,^2^ however many patients either do not respond or discontinue due to side effects. Symptoms of mania can be treated with antipsychotics and/or mood stabilisers,^2^ however these medications do not effectively address bipolar depression, neurocognitive deficits and quality of life.^3^ No significant progress has been made for decades in pharmacological treatments for BD, and polypharmacy is the norm.^4^ Faced with a lack of optimal treatments and limited biological knowledge, it is essential to use novel approaches to identify new therapeutics for BD, especially those that display novel target engagement.

The heterogeneity of BD, in combination with its complex and poorly understood neurobiology, makes it unsuitable for traditional drug discovery approaches that target a single protein thought to play a role in the aetiology and/or pathophysiology of the disease. Here we used a novel approach to drug discovery for BD to bypass this impasse, validated by our group for type 2 diabetes^5^, ^6^, ^7^ and by others in oncology.^8^ The process involves the use of next‐generation sequencing in human neuronal‐like (NT2‐N) cells to identify a gene expression signature (GES) that highlights a small number of genes whose expression levels best define the overall biological effects of a treatment or disease.

The aim of the study was to identify drugs that may be repurposed to treat BD using a GES‐based screen and then test the best candidate on depression‐like behaviours using rat models. We first created a GES for the effects of a combination of drugs commonly prescribed to treat BD, and then used the GES to screen a library of off‐patent drugs to identify those drugs that most closely mimic the effects of the BD drug combination.

## 2 MATERIALS AND METHODS

### 2.1 Ethics

Procedures were approved by the Barwon Health Human Research Ethics Committee (Project 17.205), Deakin University Human Research Ethics Committee (Project 2018‐240), North‐West University AnimCare Animal Research Ethics Committee (DoH reg. no. AREC‐130913‐015, Approval Number: NWU‐00578‐19‐A5), or Florey Animal Ethics Committee (Approval Number 20‐042‐FINMH). All procedures were concordant with the relevant national guidelines for research.

### 2.2 GES cell culture and next generation sequencing (NGS)

Ntera2 human teratocarcinoma cells (NT2) were cultured and differentiated as previously described.^9^ Following differentiation, the neuronal‐like cells (NT2‐N) were treated with the “BD drug cocktail” (2.5 mM lithium chloride, 0.5 mM valproate, 50 μM lamotrigine and 50 μM quetiapine; Sigma‐Aldrich, Castle Hill, Australia) or vehicle control (0.2% DMSO; Sigma‐Aldrich) for 24 h (*n* = 20 per group). Total RNA was extracted, quantified and quality controlled as previously described.^9^ RNAseq was performed as previously described.^9^

### 2.3 Generation of GES

NGS data was interrogated to identify the optimal set of genes (GES) that best predicts the differences between the BD drug cocktail‐treated and vehicle‐treated control cells as we have previously described.^5^ Given a set of 20 replicates per treatment, the predictive set of genes was limited to <8 to guarantee sufficient statistical estimation of joint predictors. A GES of <8 genes was also preferred in view of its subsequent requirement—a manageable number of genes that can be measured for compound library screening.

Briefly, we used diagonal linear discriminant analysis (DLDA) and the signal‐to‐noise ratio (SNR) statistic on genes with evidence of differential expression between the BD drug cocktail and vehicle‐treated cells (*p* < 0.01, *t*‐tests). DLDA was then performed with a forward stepwise variable selection to identify the minimal set of genes that best discriminate the BD drug cocktail treatment group from the vehicle treatment group. The DLDA‐selected genes were ranked using the SNR statistic according to their discriminating ability. SPSS (IBM, NY, USA) was then used to reduce signature genes to a small subset that was discriminating and displayed divergent expression profiles.

### 2.4 Compound screen

NT2‐N cells were treated with drugs (10 μM for 24 h) from the Prestwick library comprising 960 approved, off‐patent drugs, most of which have long and well‐known clinical safety histories and diverse mechanisms of action. Each plate also contained 4 wells of positive controls (drug combination) and 4 wells of negative controls (vehicle).

RNA was extracted, real‐time PCR was performed and normalised as described previously,^9^ using primers in Table S1. The GES gene expression in control samples was checked. Treatment was repeated if any gene was expressed at >2 standard deviations outside the mean for the whole screen.

The mean gene expression value for each compound was divided by the mean of the vehicle‐treated cells, and this value was used to generate an intraplate score for each compound by dividing the effect of the compound on gene expression relative to the vehicle‐treated cells by the effect of the BD drug combination relative to the vehicle. For each compound, the values of 1 minus the absolute value of the intraplate scores for each of the three genes in the GES were summed to generate an overall “similarity score” (how similar the overall effects were relative to the BD drug cocktail effects), and the compounds ranked accordingly. The net result was a ranked list of compounds in order of those that most closely resemble the effects of the BD drug combination on the GES.

### 2.5 Induced pluripotent stem cell (iPSC) generation

Blood samples were collected in EDTA tubes from a healthy adult (male, 49 years‐old, Caucasian) and peripheral blood mononuclear cells (PBMCs) were isolated from total blood cells using Lymphoprep (StemCell Technologies) in SepMate™‐15 tubes (StemCell Technologies).

PBMCs were used to generate iPSCs using Cytotune™‐iPS 2.0 Sendai Reprograming kit (ThermoFisher Scientific) at the Murdoch Children's Research Institute using a methodology described previously.^10^ The absence of mycoplasma contamination in the iPSC line was confirmed by PCR.

### 2.6 iPSC differentiation into neurons and astrocytes co‐culture

A culture of deep‐ and upper‐layer excitatory neurons and astrocytes, which form functional cortical networks, was generated from iPSCs as previously described with modifications.^11^, ^12^ Neural induction of stem cells was achieved by dual inhibition of the SMAD signalling pathway. Details of the iPSC and cortical networks' characterisation are described in Table S2.

### 2.7 Effects of trimetazidine in cortical networks

Cells were seeded (100 K/well) into 24‐well plates and treated with 0.1, 1, 10 or 100 μM trimetazidine (Sigma) or vehicle control (0.01% water). After 24 h, cells were harvested and NGS was performed as described previously. Genes with trends of up‐ or down‐regulation across increased trimetazidine doses were identified using the R package IsoGene.^13^ The likelihood ratio test for monotonic trend was calculated based on permutations (*n* = 100). *p*‐values were adjusted for multiple testing using Benjamini‐Hochberg correction.

To find potential pathways regulated by trimetazidine, genes with significant dose–response analysis results (adjusted *p*‐value<0.05) were submitted to the functional annotation tool of the database for annotation, visualization and integrated discovery (DAVID).^14^

### 2.8 Flinders sensitive line rat study

Flinders Sensitive Line (FSL) rats are a widely used genetic animal model of depression.^15^ FSL rats were bred and housed at the vivarium (SAVC reg: FR15/13458) of the North‐West University, South Africa. Male rats were group‐housed (3–4 rats/cage) from post‐natal day (PND)40, with corncob bedding changed weekly and temperature maintained at 22 ± 1°C in a relative humidity of 55% ± 10%. A 12 h light/dark cycle was followed with food provided ad libitum. Body weight was measured daily. Rats were randomly assigned into different test groups: Vehicle control, 10 mg/kg/d or 20 mg/kg/d trimetazidine (*n* = 12‐13/group), for which the doses were based on an earlier behavioural pharmacology study with trimetazidine in animals.^16^ Trimetazidine was administered in drinking water over a 28‐day period, commencing on PND40. Behavioural tests were performed as previously described in the following order: sucrose preference test on PND60, open field tests on PNDs 66 and 67, forced swim test^17^ on PND67, and elevated plus maze on PND68.^18^ Rats were humanely killed, and the hippocampi dissected and immediately immersed in isolation buffer (pH 7.2) for respiration analysis.

### 2.9 Cellular bioenergetics and mitochondrial function analysis

NT2‐N cells were seeded (80 K/well) into 24‐well Seahorse V7 plates and treated with 0.1 or 1 μM trimetazidine or vehicle control (*n* = 4) for 24 h. The oxygen consumption rate (OCR) was assessed using a Seahorse XFe Flux Analyser (Seahorse Bioscience) as previously described.^19^ Data was normalised to total protein using the Pierce BCA Protein Assay (ThermoFisher).

For respiration analyses from rat hippocampus, tissue was sampled using a 1 mM punch biopsy needle and placed in a Seahorse Islet capture plate in Kreb Henseleit Buffer (KHB) followed by capture screens before the plate was incubated in a non‐CO_2_ incubator for 10 min at 30°C. Respiration was measured for two 2 min periods, separated by a 2 min mix period, after injection of malate 5 mM, glutamate 1 mM and succinate 5 mM. 5 mM ADP (pH 7.4) was then injected and respiration was measured again. Data were normalised to total protein within each sample and the maximal respiratory capacity was established as the peak respiration values obtained following ADP injection.

### 2.10 Social isolation with chronic restraint stress rat model

To assess the effects of 30 mg/kg trimetazidine on depression‐like behaviours, adolescent rats received isolation combined with daily restraint stress. This model was chosen because it is widely used at this age,^20^ well‐established as mildly stressful to change the hypothalamus‐pituitary–adrenal axis response,^21^ and causes depression‐like behaviours that can be rescued with experimental manipulation/s.^22^, ^23^ Male Sprague–Dawley rats were weaned and group‐housed with littermates until PND 35, then weighed and moved to single‐housing in open‐top cages (60 × 40 × 26 cm) on a 12:12 h light: dark cycle (lights on:07:00). Standard chow and water were provided ad libitum. Rats were weighed every 4 days. From PND 37, each rat received restraint stress for 30 min per day for 10 days. At PND 47, rats were randomly allocated to vehicle or trimetazidine (30 mg/kg dissolved in saline with 1% DMSO) intervention administered intraperitoneally daily for 14d. At PND 59, all rats were tested in a large open field (1 × 1 meter). Rats were placed in the central zone of the arena and allowed to move freely for 10 min. At PND 60, rats were assessed in Porsolt's forced swim test, with 10 min pre‐swim learning phase in a clear perspex cylinder (10 litres) containing tepid water (23–25°C) followed by an identical 5 min test the next day (PND 61).^24^ This second session was video recorded for manual scoring for immobility, a subset of which was validated with automated and unbiased analysis of total immobility time using ForcedSwimScan (CleverSys Inc.). Trimetazidine injection occurred 5 h after behavioural tests on PND 59 and 60 (last 2 days of trimetazidine intervention) to avoid any potential acute behavioural responses to drug administration.

### 2.11 Statistical analysis

For mitochondrial respiration analyses, data were checked for normality of distribution using Kolmogorov–Smirnov tests. Because data were normally distributed and group variances were homogeneous (Levene's test), group mean differences were assessed using one‐way ANOVAs with post hoc LSD tests.

For the behavioural assessments in FSL rats, a one‐way ANOVA or Kruskal‐Wallis test was used to analyse group differences between treatment groups in the sucrose preference, open field and forced swim tests. A two‐way ANOVA was used to compare time spent in the closed and open arms of the elevated plus maze. A repeated‐measures ANOVA was used (with sphericity assumed in all instances) for bodyweight analyses. In all instances, analyses were followed by the Bonferroni post hoc test. For social isolation with chronic restraint stress rat model, *t*‐tests were used for each measure, except for bodyweight analyses that used repeated‐measures ANOVA.

## 3 RESULTS

**FIGURE 1:**
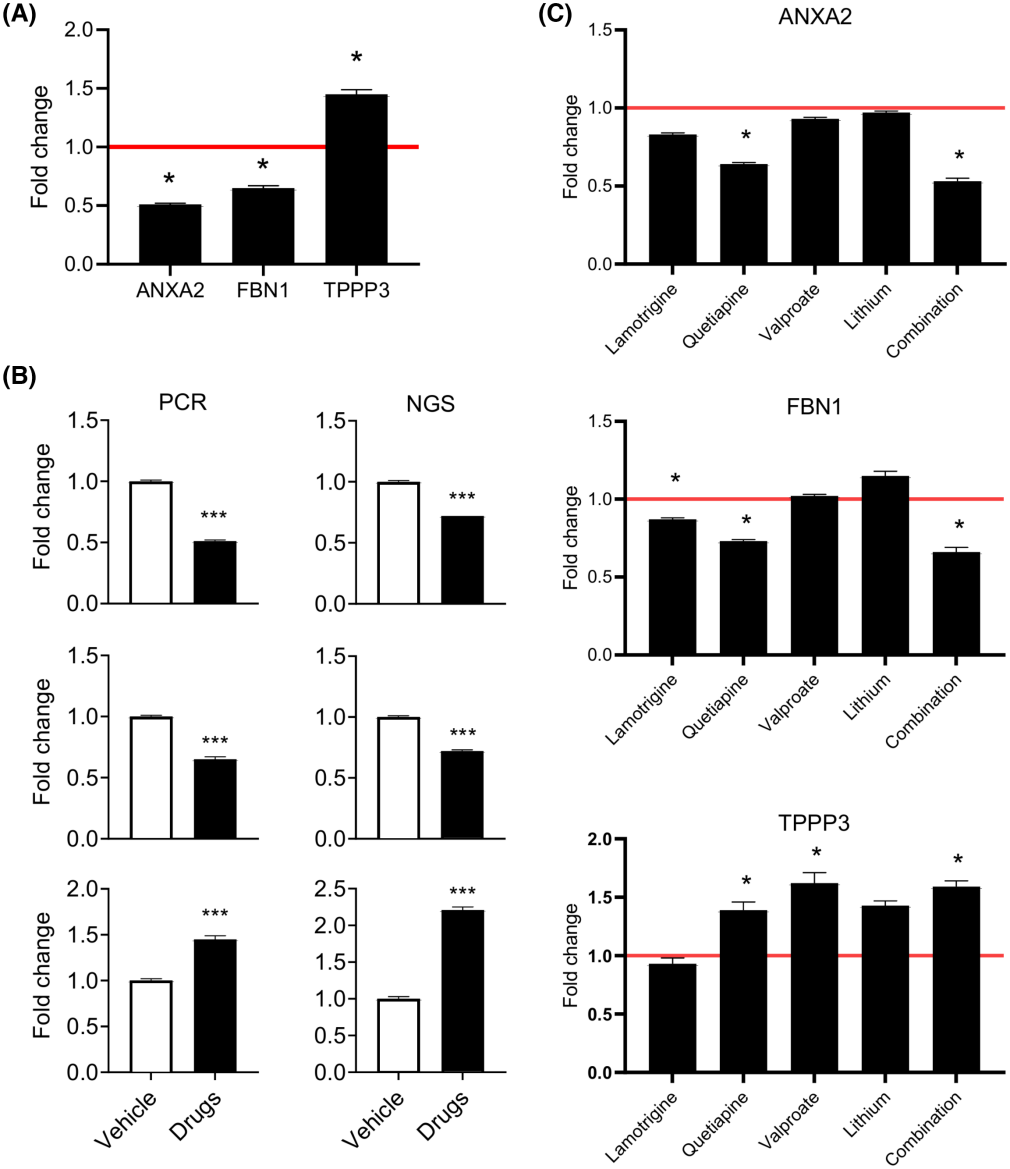
(A) Gene expression signature that best describes the overall biological effects of the bipolar disorder drug cocktail in NT2‐N cells. (B) Confirmation of the differential expression of the GES genes by RT‐PCR. (C) Confirmation of the overall effects of the four drugs on the expression of the signature genes individually and in combination (*p < 0.05; ***p < 0.001 compared to vehicle).

The overall features of the NGS dataset obtained from the NT2‐N cells treated with a combination of BD drugs are described elsewhere.^9^ A GES was generated which best describes the overall biological effects of the drug cocktail in NT2‐N cells (Figure 1A). The GES consists of 3 genes: *ANXA2* and *FBN1*, whose expression was decreased after drug treatment, and *TPP3*, which was increased. Together, the expression of these three genes predicted which treatment group the cells were in with a power of >99%. The differential expression of the GES genes was confirmed by RT‐PCR (Figure 1B). The effects of the drug cocktail on these genes were not dominated by one of the drugs but were representative of the overall effects of the 4 drugs in combination (Figure 1C), representing a net therapeutic effect.

In total, 960 drugs were screened for their effects on the expression of the GES genes in NT2‐N cells and this data was used to calculate a similarity score for each drug, with a lower similarity score indicating more similar effects to the BD drug cocktail. The screening results were filtered to exclude drugs that were not approved for use in humans, had been withdrawn or received a black box warning. The “hit” drugs from the screen are shown in Table 1, along with their effects on the expression of the GES genes and overall GES similarity scores.

**TABLE 1:** The top drugs from the gene expression screen (effects on expression of the GES genes and overall GES similarity scores).

						Fold change in expression		
Similarity Score	Name	Class	Indication	Mechanism of action	Comment	*ANXA2*	*FBN1*	*TPPP3*
0	BD drug combination	(Positive control)				0.51 +/−0.01	0.65 +/−0.02	1.45 +/−0.04
0.95	Levodopa	Dopamine precursor	Parkinson's	Increases dopamine synthesis	Side effects	0.75	0.63	1.17
1.04	Hexamethonium dibromide dihydrate	Anticholinergic	Hypertension	nicotinic cholinergic antagonist	Does not cross BBB	0.87	0.68	1.22
1.12	Clofazimine	DNA binding	Leprosy		Antibacterial	0.67	0.69	1.20
1.17	Isoxicam	NSAID	Inflammation	Anti‐inflammatory	Not novel	0.72	0.78	1.16
1.28	Harmane hydrochloride	MAO inhibitor	N/A	Neurotoxin, endogenous ligand for benzodiazepine receptor	Neurotoxic	0.56	0.68	1.09
1.30	Carteolol hydrochloride	Beta‐blocker	Glaucoma	Non‐selective beta‐adrenergic receptor antagonist; serotonin receptor antagonist	Does not cross BBB	0.63	0.66	0.97
1.34	Maprotiline hydrochloride	Antidepressant	Depression	Norepinephrine reuptake inhibitor	Not novel	0.61	0.80	1.02
1.41	Famotidine	Antihistamine	Peptic ulcer	Histamine H2 receptor antagonist		0.75	0.84	1.17
1.43	Pentolinium bitartrate	Antihypertensive	Hypertensive crisis	Nicotinic acetylcholine receptor antagonist	Unacceptable side effect profile	0.69	0.86	1.21
1.46	Mebendazole	Antiparasitic	Infection	Microtubule synthesis inhibitor	Antiparasitic	0.81	0.79	1.23
1.49	Clebopride maleate	Antiemetic	Gastrointestinal disorders	Dopamine antagonist	Extrapyramidal side effects	0.70	0.66	0.99
1.49	Cefoperazone dihydrate	Cephalosporin antibiotic	Infection	Bacterial cell wall synthesis inhibitor	Does not cross BBB	0.63	0.71	0.87
1.50	Protriptyline hydrochloride	Antidepressant	Depression/	Norepinephrine and serotonin reuptake inhibitor	Not novel	0.65	0.71	1.07
			ADHD					
1.50	Glafenine hydrochloride	NSAID	Inflammation	Anti‐inflammatory	Not novel	0.74	0.74	1.06
1.52	Pirenperone	Antipsychotic	N/A	Selective antagonist of the serotonin 5‐HT2 receptors	Not novel	1.06	0.74	1.25
1.54	Risperidone	Antipsychotic	Schizophrenia/Bipolar disorder	Dopamine antagonist and serotonin antagonist	Not novel	0.78	0.89	1.19
1.55	Trimetazidine dihydrochloride	Anti‐ischaemic	Angina pectoris	Fatty acid beta‐oxidation inhibitor		0.61	0.58	0.98
1.56	Pyrimethamine	Aminopyrimidine	Infections	Folate metabolism blocker	Antiparasitic	0.67	0.58	0.96
1.57	Benzamil hydrochloride	Sodium channel blocker	Cystic fibrosis	Inhibits renal sodium reabsorption	Does not cross BBB	0.84	0.82	1.23

**FIGURE 2:**
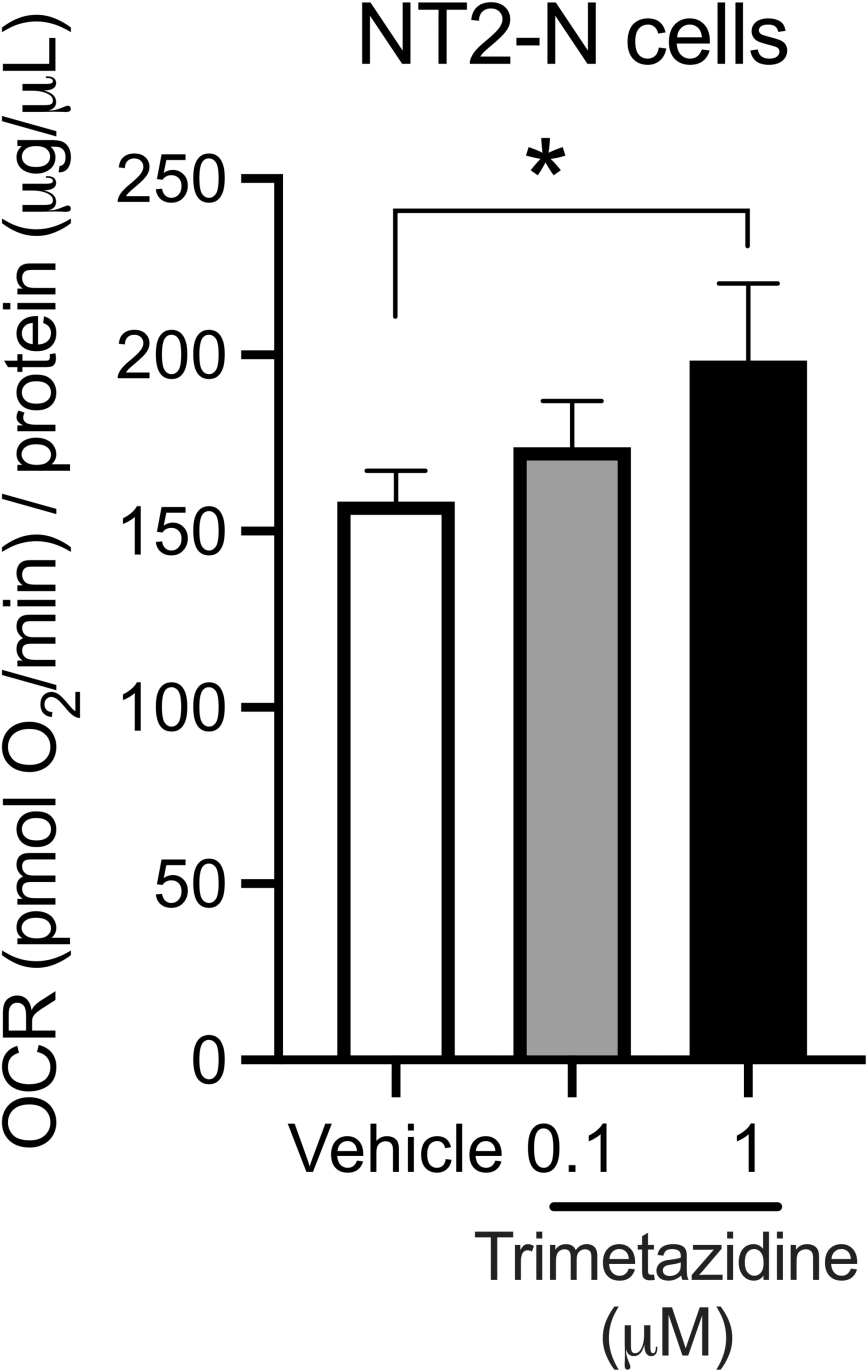
Oxygen consumption rate in NT2‐N cells after treatment with trimetazidine (*p = 0.041 compared to vehicle).

Trimetazidine was identified as a drug of interest for further investigation because it has an excellent safety profile, is known to cross the blood‐brain barrier and has not previously been used to treat neuropsychiatric disorders. Given that trimetazidine has been shown to act on mitochondrial function, we wanted to determine whether trimetazidine had a similar mechanism of action in neurons as in cardiomyocytes. Cells treated with trimetazidine displayed increased oxygen consumption indicative of improved mitochondrial function (Figure 2).

NGS was also used to investigate the transcriptional effects of trimetazidine in iPSC‐derived cortical networks, a more biologically relevant human pre‐clinical model. Isogene analysis identified several genes with evidence for dose‐dependent regulation by trimetazidine, and these genes were enriched for several pathways (Table S3), including focal adhesion and MAPK signalling. Further analysis revealed that genes in both the focal adhesion and MAPK signalling pathways showed overall evidence of downregulation as the dose of trimetazidine increased. Within the focal adhesion pathway, there were notable groups of structural genes that showed evidence of dose‐dependent downregulation by trimetazidine, including collagens, laminins and filamins (Figure S1).

**FIGURE 3:**
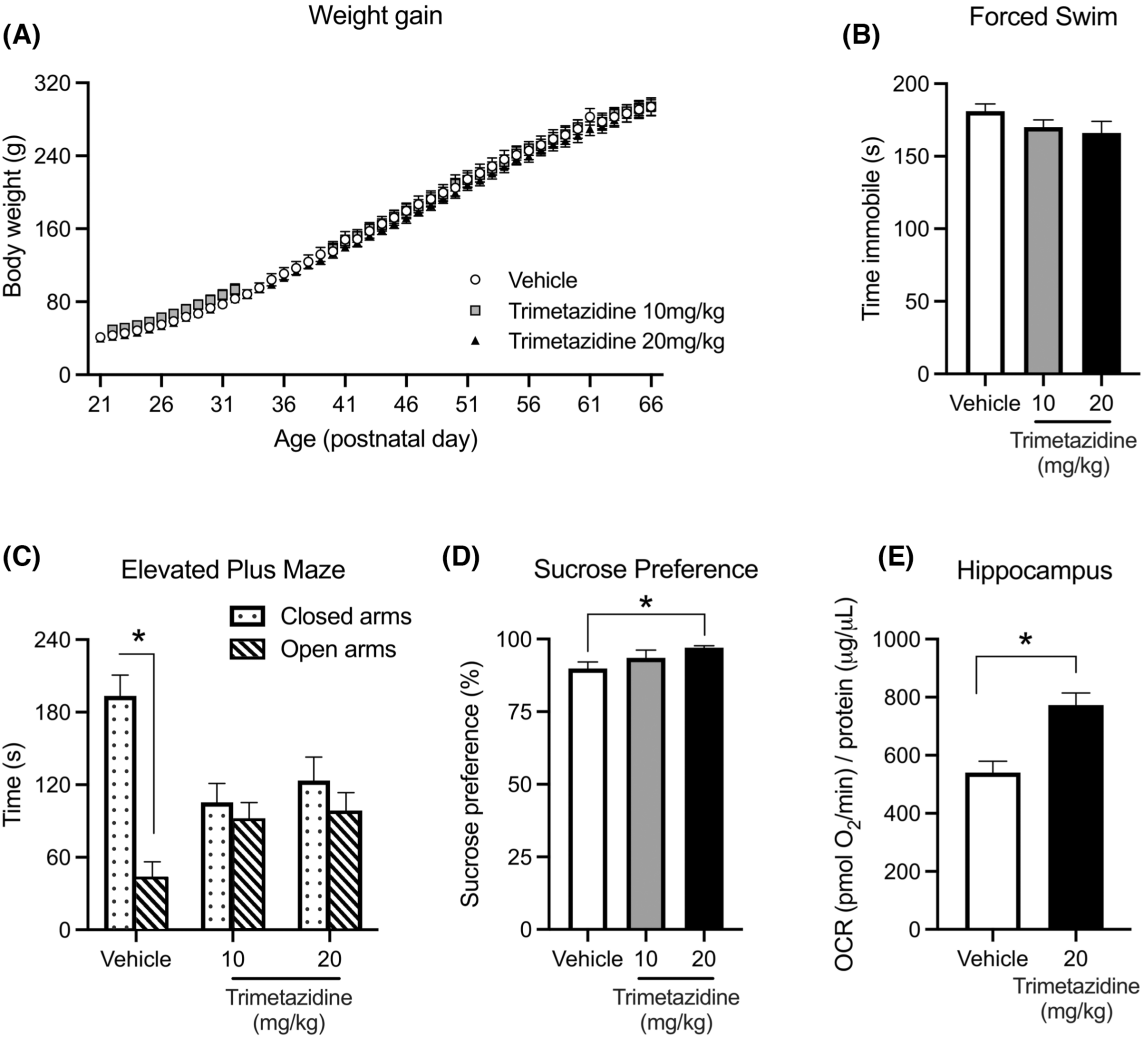
Effects of TMZ on body weight (A), depressive‐like (B), anxiety (C) and anhedonia (D) behaviours in Flinders Sensitive Line rats. (A) Flinders Sensitive Line (FSL) rats gained weight (*p* < 0.05), independent of chronic treatment with vehicle, 10 or 20 mg/kg/d trimetazidine (*p* = 0.60). (B) Chronic treatment with 10 or 20 mg/kg/d trimetazidine for 28 days tended to reduce mean time spent immobile in Flinders Sensitive Line (FSL) rats in the 5 min forced swim test (*p* = 0.11). (C) In FSL rats, mean time spent in the open arms and closed arms in an elevated plus maze (*p < 0.05 significant different between arms). (D) In FSL rats, mean percentage sucrose preference compared to vehicle (*p = 0.021). (E) Maximal respiratory capacity in hippocampus after treatment with trimetazidine (*n* = 6; *p = 0.001 compared to vehicle).

In FSL rats, treatment with 10 or 20 mg/kg/d trimetazidine for 28 days had no effect on bodyweight or general locomotor activity (Figure 3A), yet tended to reduce immobility in the forced swim test (by 8%, *p* = 0.11; Figure 3B), increase time spent in the open arms and decrease time spent in the closed arms of the EPM, consistent with an anxiolytic effect (*p* ≤ 0.0005; Figure 3C) and reduced anhedonia as indicated by increased sucrose preference at the higher dose (*p* = 0.021; Figure 3D). Trimetazidine at 20 mg/kg/d significantly increased respiratory capacity in the hippocampus of rats compared to the vehicle control group (*p* = 0.001; Figure 3E).

**FIGURE 4:**
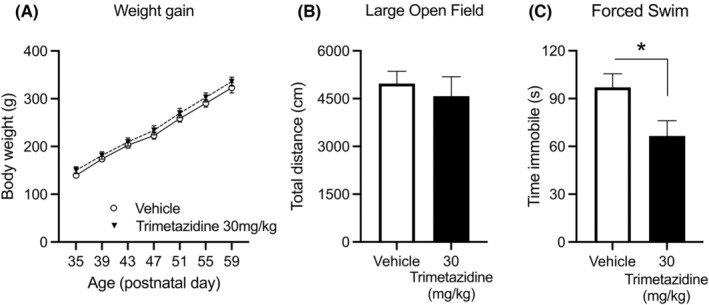
Effects of TMZ on body weight (A), anxiety (B) and depressive‐like in isolation and physical restraint rats treated with 30 mg/kg/d trimetazidine for 30 days. (A) Sprague–Dawley rats exposed to isolation and physical restraint significant gained weight (*p* < 0.0001) independent of treatment with 30 mg/kg/d trimetazidine or vehicle (Treatment: *p* = 0.34 and Treatment x Age interaction: *p* = 0.93). (B) Trimetazidine treatment had no effect on locomotion (*p* = 0.58). (C) Chronic treatment with 30 mg/kg/d trimetazidine for 14 days in isolation and physical restraint rats; mean time spent immobile in rats in the 5 min forced swim test (*p = 0.03).

Given the tendency for reduced immobility in the forced swim test in FSL rats, we conducted a follow‐up study using the social isolation with chronic restraint stress rat model of depressive‐like behaviour using a higher dose of trimetazidine (30 mg/kg/d). This study showed no effect of trimetazidine on bodyweight (*p* = 0.34) or locomotion (*p* = 0.58) (Figure 4A,B). However, trimetazidine significantly reduced immobility in the forced swim test (*p* = 0.03), consistent with an antidepressant‐like effect (Figure 4C).

## 4 DISCUSSION

A combination of transcriptomics, drug screening and in vitro and in vivo mechanistic studies identified trimetazidine as a potential drug to treat BD depression. Trimetazidine acts primarily on mitochondrial capacity, function and substrate utilisation, increasing the efficiency of ATP production, which is likely to be beneficial in BD depression. This is concordant with extant theoretical models which see mitochondrial function at the core of the disorder; decreased in depression and increased in mania with regulatory failure at the heart of the disorder.^25^ We also showed effects on focal adhesion and MAPK signalling pathways, both of which could plausibly contribute to improvements in depressive symptoms. Finally, we showed anxiolytic and antidepressant‐like properties of trimetazidine in two different animal models of depressive‐like behaviour, providing evidence to support the repurposing of trimetazidine for the treatment of BD depression.

The genes comprising the GES were: (1) *ANXA2* (Annexin 2), which encodes a calcium‐dependent phospholipid‐binding protein involved in the regulation of cellular growth and signal transduction pathways. *ANXA2* had increased gene expression in PBMCs of patients with BD (*p* < 0.001).^26^ (2) *FBN1* (Fibrillin 1), encoding an extracellular matrix protein which may be a structural component of the blood–brain barrier.^27^ Genetic variation in *FBN1* was associated with BD in a Norwegian sample (GWAS study; adj. *p* < 0.001, odds ratios 0.59–0.61).^28^ (3) *TPPP3* (Tubulin Polymerization Promoting Protein Family Member 3), which encodes a tubulin binding protein with microtubule stabilising activity and may play a role in cell proliferation and mitosis.^29^ TPPP3 has not been previously associated with BD.

The drug screen highlighted agents with known effects in the disorder such as antidepressants (maprotiline, protriptyline; both used for treating BD depression) and antipsychotics (pirenpirone, risperidone) already in use for treating mania, as well as non‐steroidal anti‐inflammatory agents (isoxicam, glafenine) which are already under investigation for such disorders.

Trimetazidine is a cytoprotective anti‐ischaemic agent used to treat stable angina pectoris. It is a selective inhibitor of 3‐ketoacyl‐CoA thiolase (the final step in beta‐oxidation of fatty acids), which results in impaired fatty acid uptake and oxidation,^30^ which in turn results in enhanced myocardial glucose oxidation, a more efficient way to make ATP.^31^ It is currently being tested for the treatment of hypertension, coronary artery disease, heart failure and hepatocellular carcinoma, and several studies/meta‐analyses have shown an overall decrease in all‐cause mortality in participants taking trimetazidine.^32^, ^33^

Mechanistic studies in cells (mainly cardiomyocytes), animal models and human participants have shown that trimetazidine improves mitochondrial function,^34^, ^35^ lowers oxidative stress^36^, ^37^ and has anti‐inflammatory properties.^38^, ^39^ All these mechanisms are currently proposed to be targets for the treatment of BD. We showed that trimetazidine has similar metabolic effects in neuronal cells, with an increase in OCR following treatment, which is consistent with previous effects seen in cardiomyocytes. This effect in cell culture translated to an improved maximal respiratory capacity in the hippocampus of rats. Therefore, the beneficial metabolic effects of trimetazidine may also be seen in the brain, and this increase in metabolic efficiency may be useful for improving symptoms in the depressive phase of BD.

We further investigated the mechanism(s) of action of trimetazidine in co‐cultured neurons and astrocytes through transcriptomic analysis. Genes affected by trimetazidine were enriched for the focal adhesion and MAPK signalling pathways, both of which appeared to be down‐regulated following treatment. Focal adhesions are specialised structures that form at cell‐extracellular matrix contact points and are involved in cell shape and motility, as well as receptor‐mediated signalling. Focal adhesion is critical for neurite outgrowth and axon pathfinding, and likely plays a key role in neuronal plasticity,^40^, ^41^ with paxilin appearing to play a central role. Among the focal adhesion genes that were downregulated, some were collagen isoforms. Overall, collagens had a strong dose‐dependent decrease in expression, which is of interest because several collagen isoforms have been identified as genetic risk factors for BD.^42^, ^43^

The MAPK signalling pathway, which regulates many processes including cell proliferation, differentiation and migration, was also transcriptionally downregulated by trimetazidine. This pathway is well known to contribute to inflammation and neuroinflammation,^44^ which is a key factor in BD pathophysiology.^45^, ^46^, ^47^ Indeed, the MAPK signalling pathway has been associated with BD in GWAS, transcriptomic and methylation studies^37^, ^39^ and MAPK signalling is increased in lymphocytes from patients with BD compared with healthy controls.^48^, ^49^ Furthermore, genetic predictors of treatment outcome in patients with BD were enriched for genes involved in the MAPK signalling pathway.^50^ Collectively, our data suggest that in addition to the predicted metabolic effects of trimetazidine, this drug may also have beneficial effects on BD symptoms by regulating the focal adhesion and MAPK signalling pathways, and identify these pathways as new targets for the treatment of BD.

From a psychotropic viewpoint, a recent animal study first demonstrated the anxiolytic properties of trimetazidine.^16^ The current study confirms and extends this, describing the broad psychotropic actions of trimetazidine, including anxiolytic, antidepressant and hedonic actions, in validated animal models of depression. This study focused on bipolar depression, but given that the drug cocktail used included mood stabilisers and drugs used to treat mania, and the screen highlighted both antidepressants and antipsychotics, future investigations should include testing the effects of trimetazidine in models of mania. In both FSL rats and rats subjected to restraint stress trimetazidine exhibited anxiolytic effects, consistent with previously published work.^16^ Trimetazidine also reduced anhedonia and tended to reduce immobility in the forced swim test at the highest dose in FSL rats, which is suggestive of antidepressant‐like properties. To investigate the latter further, we treated socially isolated rats subjected to restraint stress using a higher dose of trimetazidine and found evidence of antidepressant activity (reduced immobility in the forced swim test). Collectively these data indicate that trimetazidine has both anxiolytic and antidepressant‐like activity in rodent models of depressive behaviour and supports the repurposing of trimetazidine for the treatment of BD depression.

## CONFLICT OF INTEREST STATEMENT

The authors declare that there are no conflicts of interest.

## Supporting information


Figure S1.
**Tables S1–S3**.

## Data Availability

The data that support the findings of this study are available from the corresponding author upon reasonable request.
