# Supplementary material for: Use of a gene expression signature to identify trimetazidine for repurposing to treat bipolar depression
Source: Bipolar Disord. 2023 Mar 23;25(8):661–70. doi: 10.1111/bdi.13319 (PMC10946906; doi:10.1111/bdi.13319)

**Supplementary Table 1.** Primers used in this study.

| ***Gene*** | ***Forward primer*** | ***Reverse primer*** |
| --- | --- | --- |
| *ANXA2* | 5’-gtt tcc cgc ttg gtt gaa ca-3’ | 5’-tgt tca aag cat ccc gct ca-3’ |
| *FBN1F* | 5’-gag tgc ctt gac aat cgg ga-3’ | 5’-gat ttg gtg acg ggg ttc ct-3’ |
| *TPPP3* | 5’-tga cgg aaa gtc cgt gac ag-3’ | 5’-cct cct tgc tct tcc cct tg-3’ |

**Supplementary table 2.** Characterization of iPSCs

| **Classification** | **Test** | **Result** |
| --- | --- | --- |
| Morphology | Photography | Normal |
| Phenotype | Quantitative analysis (Flow cytometry) | 84.4 – 93.6% |
|  | Qualitative analysis (Immunofluorescence) (OCT4, SSEA4)  Real-time PCR (*OCT4, NANOG*) | Normal    Positive |
| Genotype | SNP array (resolution 0.50 Mb) | arr(1-22)x2,(XY)x1 |
| Microbiology and virology | Mycoplasma | Negative |
| Differentiation | Real-time PCR (*GFAP, MAP2, PAX6, S100B)* | Positive |
|  | Immunofluorescence (MAP2, VIM) | Normal |

**Supplementary Table 3.** Pathways altered by the treatment with trimetazidine in cortical networks.

| Pathway | Count | % | P | adj. p |  |
| --- | --- | --- | --- | --- | --- |
| Focal adhesion | 44 | 2.1 | 6.1E-06 | 0.0017 |  |
| FC gamma R-mediated phagocytosis | 21 | 1.0 | 3.2E-04 | 0.043 |  |
| Amoebiasis | 24 | 1.1 | 5.0E-04 | 0.043 |  |
| Protein processing in endoplasmic reticulum | 33 | 1.6 | 6.1E-04 | 0.043 |  |
| MAPK signalling pathway | 44 | 2.1 | 8.5E-04 | 0.049 |  |

Supplementary Figure 1


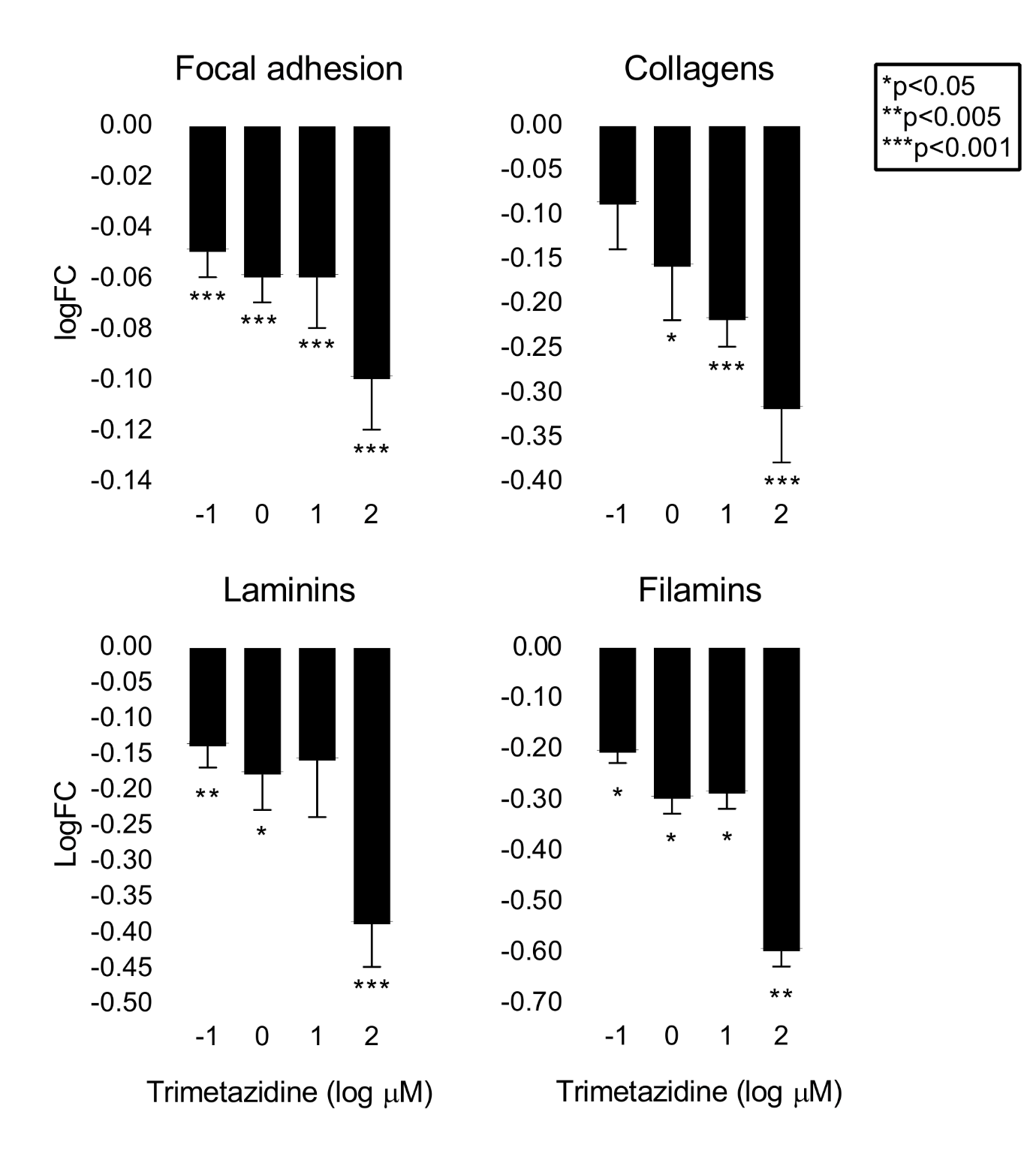

Supplement: Supplementary file 1 — Figure S1. Tables S1–S3. [file BDI-25-661-s001.docx]
